# Cation-permeable vacuolar ion channels in the moss *Physcomitrella patens*: a patch-clamp study

**DOI:** 10.1007/s00425-013-1902-4

**Published:** 2013-05-29

**Authors:** Mateusz Koselski, Kazimierz Trebacz, Halina Dziubinska

**Affiliations:** Department of Biophysics, Institute of Biology and Biochemistry, Maria Curie-Skłodowska University, Akademicka 19, 20-033 Lublin, Poland

**Keywords:** *Physcomitrella*, Potassium channels, SV channels, Tonoplast

## Abstract

Patch-clamp studies carried out on the tonoplast of the moss *Physcomitrella patens* point to existence of two types of cation-selective ion channels: slowly activated (SV channels), and fast-activated potassium-selective channels. Slowly and instantaneously saturating currents were observed in the whole-vacuole recordings made in the symmetrical KCl concentration and in the presence of Ca^2+^ on both sides of the tonoplast. The reversal potential obtained at the KCl gradient (10 mM on the cytoplasmic side and 100 mM in the vacuole lumen) was close to the reversal potential for K^+^ (*E*_K_), indicating K^+^ selectivity. Recordings in cytoplasm-out patches revealed two distinct channel populations differing in conductance: 91.6 ± 0.9 pS (*n* = 14) at −80 mV and 44.7 ± 0.7 pS (*n* = 14) at +80 mV. When NaCl was used instead of KCl, clear slow vacuolar SV channel activity was observed both in whole-vacuole and cytoplasm-out membrane patches. There were no instantaneously saturating currents, which points to impermeability of fast-activated potassium channels to Na^+^ and K^+^ selectivity. In the symmetrical concentration of NaCl on both sides of the tonoplast, currents have been measured exclusively at positive voltages indicating Na^+^ influx to the vacuole. Recordings with different concentrations of cytoplasmic and vacuolar Ca^2+^ revealed that SV channel activity was regulated by both cytoplasmic and vacuolar calcium. While cytoplasmic Ca^2+^ activated SV channels, vacuolar Ca^2+^ inhibited their activity. Dependence of fast-activated potassium channels on the cytoplasmic Ca^2+^ was also determined. These channels were active even without Ca^2+^ (2 mM EGTA in the cytosol and the vacuole lumen), although their open probability significantly increased at 0.1 μM Ca^2+^ on the cytoplasmic side. Apart from monovalent cations (K^+^ and Na^+^), SV channels were permeable to divalent cations (Ca^2+^ and Mg^2+^). Both monovalent and divalent cations passed through the channels in the same direction—from the cytoplasm to the vacuole. The identity of the vacuolar ion channels in *Physcomitrella* and ion channels already characterised in different plants is discussed.

## Introduction

The central vacuole is the largest intracellular organelle in plant cells, which stores many ions and takes part in crucial physiological processes including intracellular signalling. Different types of cation- and anion-selective channels were characterised in plant vacuoles using the patch-clamp technique (Allen and Sanders 1997; Pottosin and Schönknecht 2007; Hedrich and Marten 2011; Hedrich 2012).

The slow vacuolar channel SV is one of the best characterised vacuolar channels (Pottosin and Schönknecht 2007; Hedrich and Marten 2011; Gutla et al. 2012; Hedrich 2012). SV channels are relatively non-selective cation channels. Their permeability to K^+^ and Na^+^ is approximately equal and usually higher than that to divalent cations: Ca^2+^ and Mg^2+^. SV channels are usually activated by a high non-physiological cytoplasmic Ca^2+^ concentration, [Ca^2+^]_cyt_ exceeding 10 μM (Schulze et al. 2011). Currents passing through SV channels exhibit time-dependent saturation and rectification promoting cation transport from the cytosol to the vacuolar lumen. The membrane potential activating SV currents is shifted to positive values in respect to a physiological (negative) potential range. These two factors seem to limit the physiological role(s) of SV channels to osmoregulation and sequestration of toxic cations. On the other hand, multiple ways of SV channel regulation by factors that are usually lost during vacuole isolation for a patch-clamp study, like kinases/phosphatases, 14-3-3-proteins, polyamines, etc., make the question of their role in cell signalling open. Identification of the gene encoding the SV channel protein (Peiter et al. 2005) allowed quick progress in examination of SV channel operation (Isayenkov et al. 2010; Dadacz-Narloch et al. 2011; Schulze et al. 2011). Subtle structural differences in SV channels among different plants in connection with the multiple steps of their regulation cause significant differences in their kinetics, susceptibility to activating factors, and surface density (Rienmüller et al. 2010).

Potassium cations, most abundant in the vacuole, can also flow through two different vacuolar channels: highly selective potassium channels (VK) and fast-activated channels (FV). VK channels were originally found in guard cell vacuoles (Ward and Schroeder 1994) and then in other plant tissues (Pottosin et al. 2003). They are activated by [Ca^2+^]_cyt_ in a micromolar range. VK channels are voltage-independent. In *Arabidopsis*, VK channels are encoded by *TPK1*—a tandem pore channel (Gobert et al. 2007).

FV channels are non-selective cation channels (Allen et al. 1998; Pottosin et al. 2003). In contrast to SV channels, they activate instantaneously. FV channels are active at a submicromolar Ca^2+^ concentration. Higher concentrations of these cations inhibit FV channels on both sides of the vacuolar membrane (Tikhonova et al. 1997). Such a great variety of vacuolar potassium channels indicates that the vacuole plays a crucial role in K^+^ homeostasis.

The moss *Physcomitrella patens* is a model plant often used by molecular biologists and geneticists due to its highly efficient homologous recombination, allowing generation of knockout mutants (by means of gene targeting) and testing functions of selected genes. *Physcomitrella* is a well-known model for research of the impact of some environmental factors on physiological processes in plants. Up to now, the effects of salinity, drought and osmotic stress (Frank et al. 2005), nutritious element deficiency (Kroemer et al. 2004), and low and high temperature have been examined (Minami et al. 2005; Saidi et al. 2009).

The *TPC1* ortholog from *Physcomitrella patens* was expressed in *Arabidopsis thaliana* with silenced native *TPC1* genes encoding SV channel proteins (Dadacz-Narloch et al. 2011). Clear SV channel activity was demonstrated in this heterologous system. However, up to now, there are no published electrophysiological data concerning vacuolar ion channels in *Physcomitrella* wild-type plants. We decided to fill this gap and examine ion channels of *Physcomitrella* in a native system—vacuoles isolated from the moss gametophyte.

## Materials and methods

### Plant material

The moss *Physcomitrella patens* was a kind gift from Prof. Dr. Ralf Reski (University of Freiburg, Germany). Moss gametophytes were cultivated in a growth chamber at 22 °C under a 16/8 h light/dark photoperiod with light intensity of 60 μmol m^−2^ s^−1^ in Petri dishes containing solidified Knop medium (Reski and Abel 1985). Maintenance of the moss culture was achieved by monthly sub-culturing of young gametophytes into a fresh medium. The fast rate of growth and great abilities of vegetative reproduction resulted in formation of a small colony of a few young gametophytes from one gametophyte after 2–3 weeks.

### Vacuole isolation

Vacuole isolation was carried out using a nonenzymatic method described by Trebacz and Schönknecht (2000). Before the experiments, the gametophyte leaves were plasmolysed in a medium containing 650 mM sorbitol, 15 mM HEPES/Tris, pH 7, and different ions, depending on the type of the experiment. After 20–30 min, the leaves were cut with a razor blade and transferred to the measuring chamber containing a medium with a slightly lower osmotic pressure than the plasmolysing solution. During deplasmolysis, a few protoplasts that were not destroyed by incision emerged in the cutting line from open cell walls. Reduction of the osmotic pressure of the medium to 300 mOsm kg^−1^ resulted in rupture of the protoplasts and vacuole release.

### Patch-clamp experiments

Currents flowing either through the whole tonoplast (“whole-vacuole” configuration) or through a small patch of the tonoplast (“cytoplasm-out” configuration) were measured during the patch-clamp experiments. The micropipettes were made of borosilicate glass tubes with 1.5 mm outer diameter (Kimax-51, Kimble Products) pulled using a two-step puller PP-830 (Narishige, Tokyo, Japan) and fire-polished by a microforge MF 200-2 (World Precision Instruments, Sarasota, FL, USA). An Ag/AgCl reference electrode with a ceramic porous bridge was immersed in the bath solution and filled with 100 mM KCl. Liquid junction potentials were calculated using JPCalcW written by P.H. Barry (University of New South Wales, Sydney, Australia). Osmolality of all solutions used during the measurements was adjusted with sorbitol to 300 mOsm kg^−1^ using a cryoscopic osmometer (Osmomat 030, Gonotec). Recordings were made using a patch-clamp amplifier EPC-10 (Heka Electronik) running under the Patchmaster software (Heka Elektronik). The sampling rate was 10 kHz and filter frequency was 2 kHz. Statistical analysis, current/voltage characteristics (I/V), and current density/voltage characteristics (J/V) were elaborated using SigmaPlot 9.0 (Systat Software Inc.). Current and voltage signs followed the convention proposed by Bertl et al. (1992).

### Solution composition

Basic solutions used to examine the activity of K^+^-permeable channels contained 100 mM KCl, 2 mM CaCl_2_, 15 mM HEPES/Tris, pH 7 in the bath (cytosol) and 100 mM KCl, 2 mM CaCl_2_, 15 mM MES/Tris, pH 5.8 in the pipette (vacuole lumen). Solutions used to examine the activity of Na^+^-permeable channels were composed of 100 mM NaCl, 2 mM CaCl_2_, 15 mM HEPES/Tris, pH 7 in the bath and 100 mM NaCl, 2 mM CaCl_2_, 15 mM MES/Tris, pH 5.8 in the pipette. Selectivity of K^+^- and Na^+^-permeable channels was checked by changing solutions in the bath to 10 mM KCl, 2 mM CaCl_2_, 15 mM HEPES/Tris, pH 7 (in the case of K^+^-permeable channels) and 10 mM NaCl, 2 mM CaCl_2_, 15 mM HEPES/Tris, pH 7 (in the case of Na^+^-permeable channels). Dependence of the channel activity on Ca^2+^ was checked using different concentration of that ion. The concentration of free Ca^2+^ was determined using a Ca-EGTA Calculator v1.2 (http://maxchelator.stanford.edu/CaEGTA-NIST.htm). Solutions used to study the activity of Mg^2+^-permeable channels consisted of 10 mM MgCl_2_, 15 mM HEPES/Tris, pH 7 in the bath and 100 mM MgCl_2_, 15 mM MES/Tris, pH 5.8 in the pipette. Solutions used to study the activity of Ca^2+^-permeable channels consisted of 1 mM CaCl_2_, 15 mM HEPES/Tris, pH 7 in the bath and 10 mM CaCl_2_, 15 mM MES/Tris, pH 5.8 in the pipette. Exchange of the solutions in the measuring chamber was accomplished by a peristaltic pump (Ismatec ISM796B) before recording.

## Results

We examined ion channels in *P.* *patens* in symmetrical (in the bath and in the pipette) solutions containing 100 mM KCl and 2 mM CaCl_2_. These conditions favoured activation of SV channels in *Conocephalum conicum*—a bryophyte closely related to *Physcomitrella* (Trebacz and Schönknecht 2000).

**Fig. 1:**
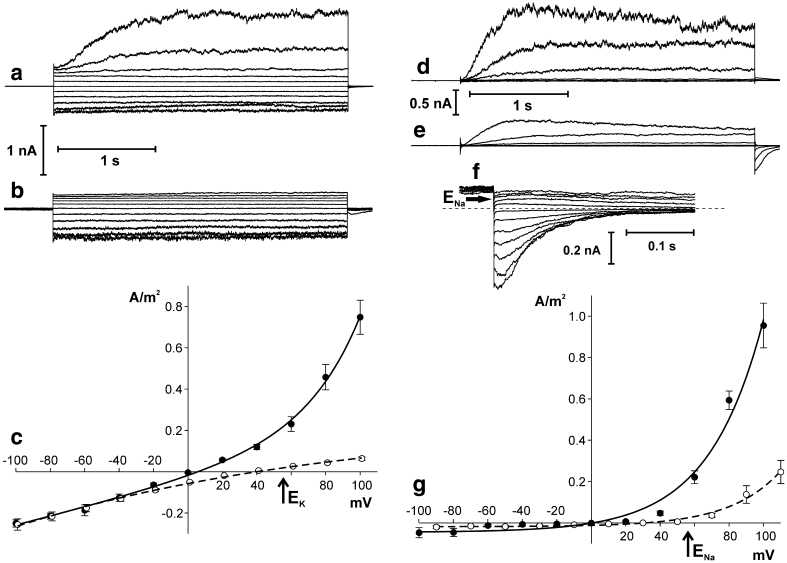
Ion channel activity recorded in *Physcomitrella patens* vacuoles when the current was carried by K^+^ (**a**–**c**) and Na^+^ (**d**–**g**). **a** Whole-vacuole currents recorded at 100 mM KCl, 2 mM CaCl_2_, 15 mM HEPES/Tris, pH 7 in the medium and 100 mM KCl, 2 mM CaCl_2_, 15 mM MES/Tris, pH 5.8 in the pipette. **b** Whole-vacuole currents recorded at 10 mM KCl, 2 mM CaCl_2_, 15 mM HEPES/Tris, pH 7 in the medium and 100 mM KCl, 2 mM CaCl_2_, 15 mM MES/Tris, pH 5.8 in the pipette. **c** J–V curves obtained in the same conditions as in **a** (*solid line* and *closed circles*, *n* = 6) and **b** (*dashed lines* and *open circles*, *n* = 10), respectively. **d** Whole-vacuole currents recorded at 100 mM NaCl, 2 mM CaCl_2_, 15 mM HEPES/Tris, pH 7 in the medium and 100 mM NaCl, 2 mM CaCl_2_, 15 mM MES/Tris, pH 5.8 in the pipette. **e** Whole-vacuole currents recorded at 10 mM NaCl, 2 mM CaCl_2_, 15 mM HEPES/Tris, pH 7 in the medium and 100 mM NaCl, 2 mM CaCl_2_, 15 mM MES/Tris, pH 5.8 in the pipette. **f** Tail currents recorded in the same conditions as in **e**. Holding voltage (90 mV) lasted 2 s and then test voltages from −30 to 80 mV with 10 mV steps were applied. *Dashed line* indicates zero current. *Horizontal arrow* points to the reversal potential. **g** J–V curves obtained in the same conditions as in **d** (*solid line* and *closed circles*, *n* = 4) and **e** (*dashed lines* and *open circles*, *n* = 4), respectively (*n* denotes different vacuoles). Whole-vacuole recordings were obtained by application of 0.5 s holding voltage (0 mV for **a**, **d**, 1 mV for **b** and 10 mV for **e**), then 3 s test voltages with 20 mV steps (from 100 to −100 mV for **a**, **d**, from 101 to −99 mV for **b** and from 110 to –90 mV for **e**), and 0.3 s pulse (0 mV for **a**, **d**, 1 mV for **b** and 10 mV for **e**) after test voltage

The whole vacuole records in *Physcomitrella* (Fig. 1a, b) differed from typical SV. In addition to slowly activating currents at positive voltages, fast activating currents were recorded at both positive and negative tonoplast voltages. The density of the currents was higher at positive than at negative voltages (Fig. 1c). The tenfold decrease in the K^+^ concentration to 10 mM on the cytoplasmic side caused shift of the reversal potential close to* E*_K_ indicating K^+^ selectivity. In addition, the KCl gradient caused a decrease in positive currents (Fig. 1b, c). A decrease in the K^+^ concentration on the cytoplasmic side did not change negative currents, which could be carried by other than the SV channel type.

Application of Na^+^ instead of K^+^ in the bath and pipette solutions revealed that the fast-activated component of the currents disappeared leaving SV currents unchanged (Fig. 1d). Currents passing through SV channels decreased after a tenfold decrease in the Na^+^ concentration to 10 mM on the cytoplasmic side (Fig. 1e, g). In such conditions, relaxation currents (tail currents) were observed (Fig. 1f). The reversal potential for those currents was at positive voltages close to *E*_Na_. A positive shift of the reversal potential was also observed at J–V curves calculated from the steady state currents (Fig. 1g). These results confirmed, on the one hand, nearly equal permeability of SV channels to K^+^ and Na^+^ and, on the other hand, impermeability of fast-activated channels to Na^+^, which points to K^+^ selectivity of these channels.

**Fig. 2:**
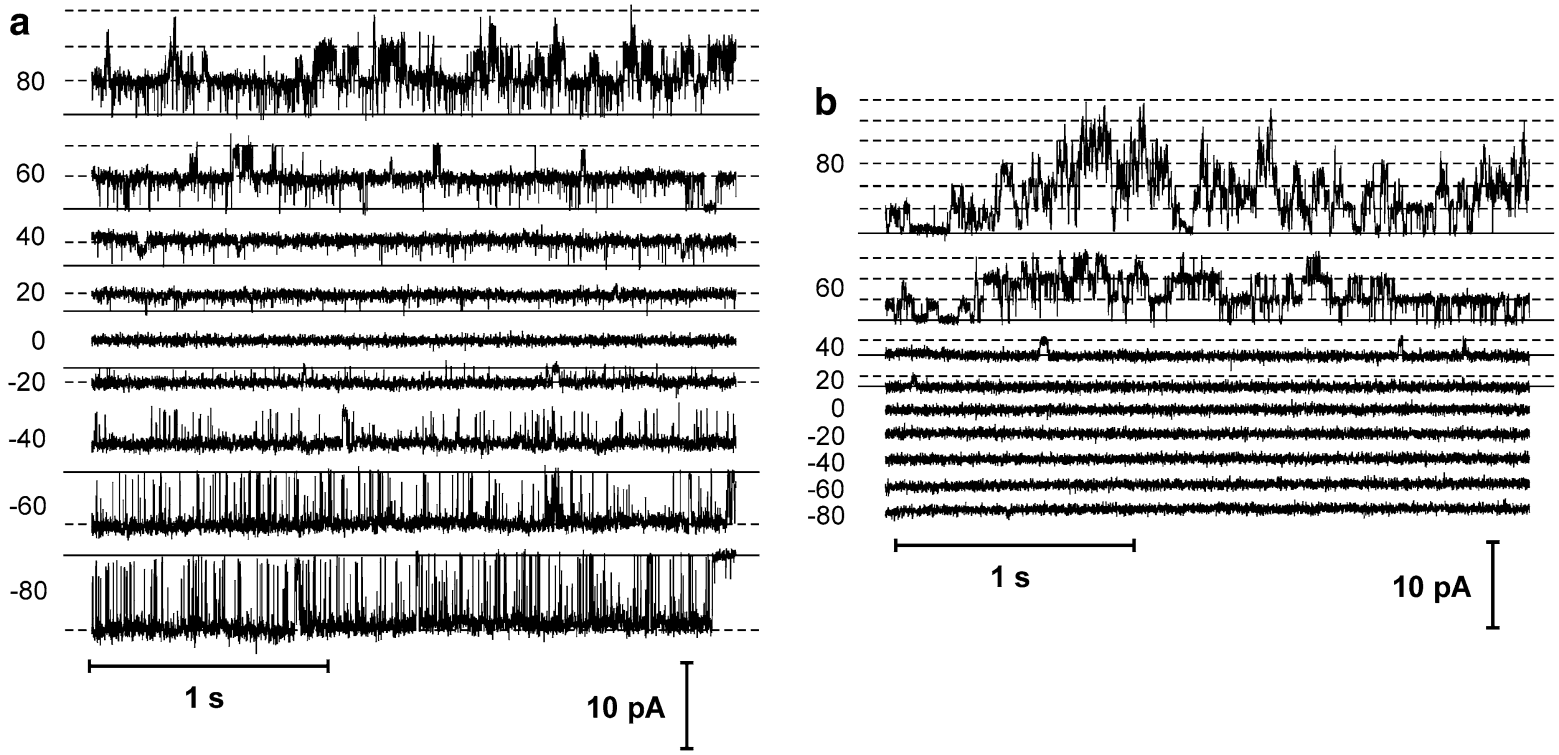
Single channel activity recorded in *Physcomitrella patens* vacuolar patches when the current was carried by K^+^ (**a**) and Na^+^ (**b**). **a** The cytoplasm-out currents recorded at 100 mM KCl, 2 mM CaCl_2_, 15 mM HEPES/Tris, pH 7 in the medium and 100 mM KCl, 2 mM CaCl_2_, 15 mM MES/Tris, pH 5.8 in the pipette. **b** The cytoplasm-out currents recorded at 100 mM NaCl, 2 mM CaCl_2_, 15 mM HEPES/Tris, pH 7 in the medium and 100 mM NaCl, 2 mM CaCl_2_, 15 mM MES/Tris, pH 5.8 in the pipette

The activity of single channels was recorded in isolated tonoplast patches in the cytoplasm-out configuration excised from vacuoles in whole-vacuole configuration. Recordings obtained in the symmetrical K^+^ concentration (as in Fig. 1a) show the activity of the channels at both negative and positive voltages (Fig. 2a). The unitary conductance of the channels was higher at negative voltages [91.6 ± 0.9 pS (*n* = 14) at −80 mV] than at positive ones [44.7 ± 0.7 pS (*n* = 14) at +80 mV]. The reason for the difference in conductance could be either the activity of two different types of channels or the multi-ion character of one channel allowing simultaneous conductance of more than one ion. SV channels in different plant vacuoles are reported to possess such characteristics. However, the open probability of these channels at negative voltages is very low. In contrast, in *Physcomitrella* the open probability at negative voltages was very high—it reached 0.946 ± 0.005 (*n* = 4) at −80 mV (in conditions as in Fig. 2a). These results indicate once more that at negative voltages currents passing through channels different from SV have been recorded.

In order to separate SV currents from the others, we replaced K^+^ with Na^+^ on both sides of the tonoplast. In such conditions, we recorded channel activity only at positive voltages with characteristic time dependence of SV channels (Fig. 2b). Conductance of this channel was smaller than that of the channel examined in the symmetrical K^+^ concentration, and at +80 mV it reached 30.9 ± 0.5 pS (*n* = 6).

**Fig. 3:**
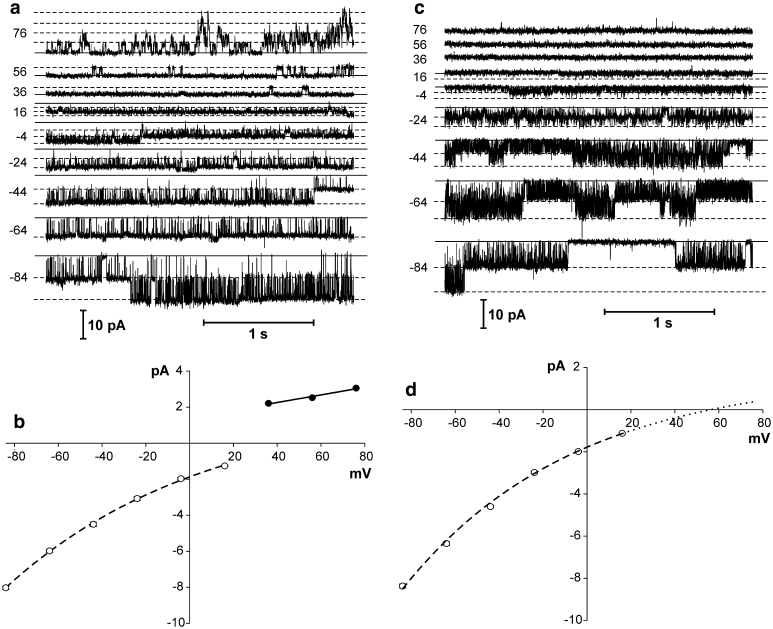
Simultaneous activity of two types of channels in *Physcomitrella patens* vacuolar patches. Recordings obtained when the currents were carried by Na^+^ (positive currents—SV type channels) and K^+^ (negative currents—K^+^-selective channels). **a** The cytoplasm-out currents recorded at 100 mM NaCl, 10 μM CaCl_2_, 15 mM HEPES/Tris, pH 7 in the medium and 100 mM KCl, 2 mM CaCl_2_, 15 mM MES/Tris, pH 5.8 in the pipette. **b** I–V curves obtained in the same conditions as in **a** (*n* = 5). *Solid line* and *closed circles* denote currents carried by SV type channels and *dashed line* and *open circles*—K^+^-selective channels. **c** Cytoplasm-out currents recorded at 100 mM NaCl, 0.1 μM CaCl_2_, 15 mM HEPES/Tris, pH 7 in the medium and 100 mM KCl, 2 mM EGTA, 15 mM MES/Tris, pH 5.8 in the pipette. **d** I–V curve obtained in the same conditions as in **c** (*n* = 7) (*n* denotes different vacuoles)

Application of Na^+^ on the cytoplasmic side of the membrane and K^+^ on the vacuole lumen allowed separation of two types of currents (Fig. 3a, b). Under such conditions, SV channels could be active at both positive and negative voltages, whereas K^+^-selective channels only at negative voltages. The I–V curves (Fig. 3b) show Na^+^ currents flowing through SV channels (solid regression line) and K^+^ currents (dashed regression line) flowing through K^+^-selective channels. The decrease in [Ca^2+^]_cyt_ to 0.1 μM caused a decrease in the open probability of the SV channels but not of the K^+^-selective channels (Fig. 3c, d). The results indicate that the K^+^-selective, rather than SV channels, require lower [Ca^2+^]_cyt_ to be active.

**Fig. 4:**
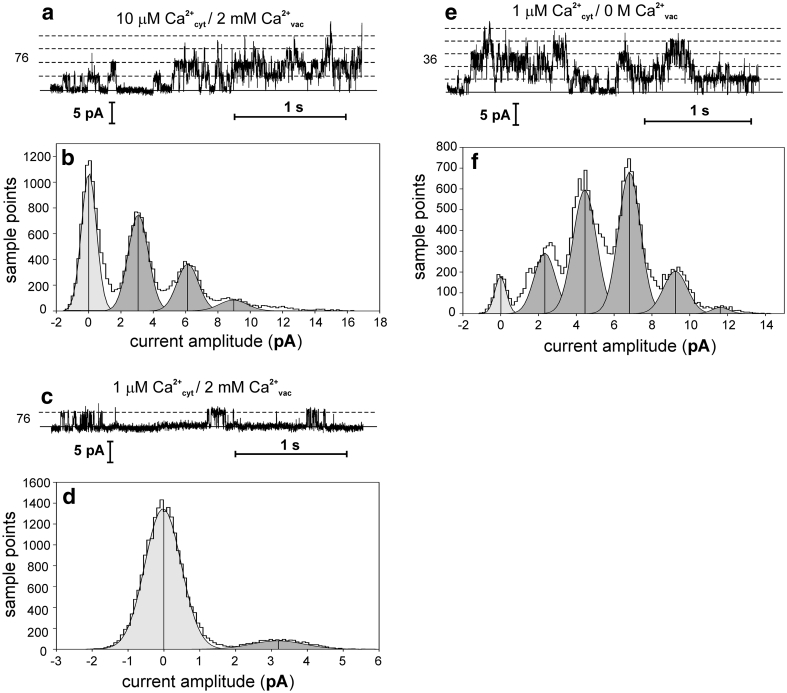
Influence of cytoplasmic and vacuolar calcium on the SV type channel activity. The records were obtained in the cytoplasm-out configuration at 76 mV (**a**, **c**) and 36 mV (**e**). **a** Channel activity recorded at 10 μM CaCl_2_, 100 mM NaCl, 15 mM HEPES/Tris, pH 7 in the medium and 2 mM CaCl_2_, 100 mM KCl, 15 mM MES/Tris, pH 5.8 in the pipette. **b** Histogram based on four records as in **a**. **c** Channel activity recorded at 1 μM CaCl_2_, 100 mM NaCl, 15 mM HEPES/Tris, pH 7 in the medium and 2 mM CaCl_2_, 100 mM KCl, 15 mM MES/Tris, pH 5.8 in the pipette. **d** Histogram based on four records as in **c**. **e** Channel activity recorded at 1 μM CaCl_2_, 100 mM NaCl, 15 mM HEPES/Tris, pH 7 in the medium and 2 mM EGTA, 100 mM KCl, 15 mM MES/Tris, pH 5.8 in the pipette. **f** Histogram based on four records as in **e**

Dependence of SV channel activity on the cytoplasmic Ca^2+^ concentration was further examined using 10 μM [Ca^2+^]_cyt_ (Fig. 4a, b), and 1 μM [Ca^2+^]_cyt_ (Fig. 4c, d) at a high vacuolar Ca^2+^ concentration (2 mM). The histograms based on the recordings carried out at different [Ca^2+^]_cyt_ show that the decrease in [Ca^2+^]_cyt_ caused a decrease in the open probability but did not affect conductance. Not only the cytoplasmic, but also vacuolar Ca^2+^ concentration [Ca^2+^]_vac_ affected SV channel activity. While [Ca^2+^]_cyt_ activated the SV channels, [Ca^2+^]_vac_ inhibited their activity. Measurements carried out at [Ca^2+^]_vac_ reduced to zero and 1 μM [Ca^2+^]_cyt_ (Fig. 4e, f) showed that there was high activity of SV channels even at +36 mV. The inhibitory effect of [Ca^2+^]_vac_ on SV channel activity is a common phenomenon in vascular plants. It was observed, among others, in *Beta vulgaris* (Pottosin et al. 2004) and *A. thaliana* (Beyhl et al. 2009).

**Fig. 5:**
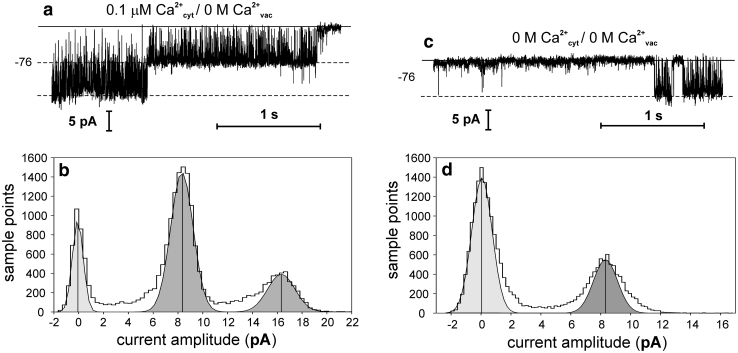
Influence of cytoplasmic calcium on the K^+^-selective channel activity. The records were obtained in the cytoplasm-out configuration at −76 mV. **a** Channel activity recorded at 0.1 μM CaCl_2_, 100 mM NaCl, 15 mM HEPES/Tris, pH 7 in the medium and 2 mM EGTA, 100 mM KCl, 15 mM MES/Tris, pH 5.8 in the pipette. **b** Histogram based on four records as in **a**. **c** Channel activity recorded at 2 mM EGTA, 100 mM NaCl, 15 mM HEPES/Tris, pH 7 in the medium and 2 mM EGTA, 100 mM KCl, 15 mM MES/Tris, pH 5.8 in the pipette. **d** Histogram based on four records as in **c**

Apart from the SV channels, also the K^+^-selective channels proved to be calcium dependent (Fig. 5a–d). In comparison to the SV channels, the K^+^-selective channels were active even in the absence of Ca^2+^ on both sides of the tonoplast (Fig. 5c, d). However, the open probability of the channels in the absence of Ca^2+^ on the cytoplasmic side was low and amounted to 0.302 ± 0.105 (*n* = 4). At low physiological [Ca^2+^]_cyt_ equal 0.1 μM, the K^+^-selective channels exhibited high activity (Fig. 5a, b). The open probability equalled 0.674 ± 0.042 (*n* = 4).

**Fig. 6:**
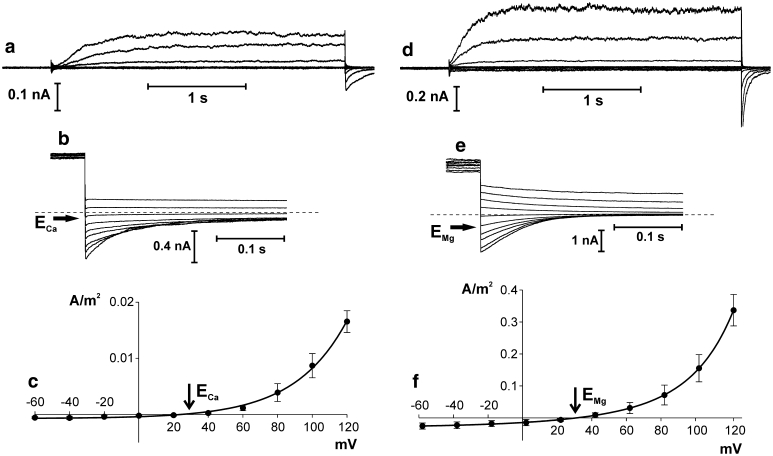
Ion channel activity recorded in *Physcomitrella patens* vacuoles when the currents were carried by Ca^2+^ (**a**–**c**) and Mg^2+^ (**d**–**f**). **a** Whole-vacuole currents recorded at 1 mM CaCl_2_, 15 mM HEPES/Tris, pH 7 in the medium and 10 mM CaCl_2_, 15 mM MES/Tris, pH 5.8 in the pipette. **b** Tail currents recorded at 5 mM CaCl_2_, 15 mM HEPES/Tris, pH 7 in the medium and 50 mM CaCl_2_, 15 mM MES/Tris, pH 5.8 in the pipette. Holding voltage (100 mV) lasted 2 s and then test voltages from −30 to 50 mV with 10 mV steps were applied. *Dashed line* indicates zero current. *Horizontal arrow* points to the reversal potential. **c** J–V curve obtained in the same conditions as in **a** (*solid line* and *closed circles*, *n* = 4). **d** Whole-vacuole currents recorded at 10 mM MgCl_2_, 15 mM HEPES/Tris, pH 7 in the medium and 100 mM MgCl_2_, 15 mM MES/Tris, pH 5.8 in the pipette. **e** Tail currents recorded in the same conditions as in **d**. Holding voltage (102 mV) lasted 2 s and then test voltages from −8 to 82 mV with 10 mV steps were applied. *Dashed line* indicates zero current. *Horizontal arrow* points to the reversal potential. **f** J–V curve obtained in the same conditions as in **d** (*n* = 7) (*n* denotes different vacuoles). Whole-vacuole recordings were obtained by application of 0.5 s holding voltage (20 mV for **a**, and 22 mV for **d**), then 3 s test voltages with 20 mV steps (from 120 to −60 mV for **a** and from 122 to −58 mV for **d**), and 0.3 s pulse (20 mV for **a** and 22 mV for **d**) after test voltage

Since SV channels in almost all plant species tested so far are permeable to both mono- and divalent cations, it was important to record whole vacuole currents in the presence of Ca^2+^ as the only permeable cation. Whole vacuole recordings in the Ca^2+^ gradient promoting Ca^2+^ efflux from the vacuole showed typical SV currents (Fig. 6a). The reversal potential obtained from relaxation currents (Fig. 6b) and from steady state currents (Fig. 6c) was close to *E*_Ca_. The density of the currents was very low (Fig. 6c) and no significant current appeared at negative voltages, indicating negligible Ca^2+^ flow from the vacuole to the cytoplasm. Figure 6 presents also the results of SV channel activity when Mg^2+^, another divalent cation, was the only permeable ion. Permeability of the channels to Mg^2+^ was confirmed by the reversal potential located close to *E*_Mg_ (Fig. 6e, f). The whole-vacuole currents (Fig. 6d) and current densities (Fig. 6f) show that Mg^2+^, likewise Ca^2+^, flew through the tonoplast mostly in one direction—from the cytoplasm to the vacuole.

## Discussion

Potassium is the main ion present in the vacuole. This ion can flow through the tonoplast by different types of ion channels (Allen and Sanders 1997; Pottosin et al. 2003; Pottosin and Schönknecht 2007; Hedrich and Marten 2011; Hedrich 2012). Among them is the SV channel, whose activity has been recorded in the tonoplast of all higher plants tested (Pottosin et al. 1997), but not in Charophyta algae (Laver and Walker 1991; Linz and Köhler 1994). Activity of these ion channels has also been recorded in the liverwort *Conocephalum conicum* phylogenetically related to *Physcomitrella patens* (Trebacz and Schönknecht 2000). Recent experiments have demonstrated the existence of SV channels in *Physcomitrella patens*. The *TPC1* ortholog from *Physcomitrella* expressed in *Arabidopsis* gave typical SV currents (Dadacz-Narloch et al. 2011).

In the vacuoles isolated from *Physcomitrella* (in a native system), we recorded complex currents in conditions favourable for activation of SV channels. The whole-vacuole currents in the symmetrical KCl concentration differed in kinetics depending on the tonoplast polarisation. At positive voltages, currents with slow kinetics of activation were recorded. At negative and partially at positive voltages, currents passing through the *Physcomitrella* tonoplast exhibited fast activation and values exceeding by orders of magnitude the SV currents at these voltages (Fig. 1a). Occasionally, clear SV currents were recorded in these conditions with practically no currents at negative voltages (data not shown). Thus, it can be concluded that in most cases we registered currents passing through two different types of ion channels. These could be two different K^+^-conducting channels including SV, or SV and an anion channel conducting Cl^−^. A similar result was registered in vacuoles of *Conocephalum conicum* where at a high [Ca^2+^]_cyt_ concentration SV and anion channels were activated at positive and negative voltages, respectively, both nearly perfectly rectifying—promoting K^+^ and Cl^−^ flux from the cytosol to the vacuole (Trebacz et al. 2007). However, application of the KCl gradient favouring K^+^ and Cl^−^ flux from the vacuole to the cytosol should reduce both currents. This was not the case at negative voltages (Fig. 1b, c), which makes activation of anion channels questionable. Moreover, the reversal potential of whole-vacuole currents determined upon the KCl gradient being close to *E*_K_ practically eliminates anion channels from consideration.

The open probability of SV channels in different plant species is much higher at positive than at negative voltages (in the symmetrical KCl concentration). The high and nearly equal open probability at positive and negative voltages recorded in the symmetrical concentration of 100 mM KCl and 2 mM CaCl_2_ (*P*_o_ = 0.961 ± 0.014 at +80 mV and *P*_o_ = 0.946 ± 0.005 at −80 mV) (Fig. 2a) indicates that at negative voltages activity of channels different from SV is recorded. After blockage of negative but not positive currents by Na^+^, it was almost clear that apart from cation-selective SV channels active mostly at positive voltages, channels that distinguish K^+^ over Na^+^ are active. Existence of two different channels possessing different selectivity was also confirmed using Na^+^ in the cytoplasm and K^+^ in the vacuolar lumen as main permeable ions (Fig. 3).

Measurements with different [Ca^2+^]_cyt_ showed differences in the calcium dependence of SV and K^+^-selective channels (Figs. 4, 5). Dependence of vacuolar cation channel activity on [Ca^2+^]_cyt_ occurs for example in *Vicia faba,* where changes in [Ca^2+^]_cyt_ caused changes in activation of three different cation-permeable channels: SV, VK, and FV (Allen and Sanders 1996). Ca^2+^-dependence could be a good criterion of identifying channel types operating in the *Physcomitrella* vacuole. In addition to SV channels, activation of two other channels in *Physcomitrella* needs consideration, i.e. FV and VK channels. FV channels are relatively non-selective cation-permeable transporters (Brüggemann et al. 1999) which require a [Ca^2+^]_cyt_ level of approx. 10^−7^ M for activation, which is much lower than in the case of SV channels. FV channels in vascular plants exhibit rectification (Tikhonova et al. 1997), which was not the case in *Physcomitrella* (Figs. 1a, c,  2a). Another feature that allowed us to exclude FV channels from consideration was impermeability of the *Physcomitrella* channels to Na^+^. It is much more probable that we recorded currents passing through VK channels which are highly K^+^-selective and do not possess rectification abilities (Isayenkov et al. 2010).

The vacuolar membrane of *Physcomitrella* is permeable not only to K^+^ but also to divalent cations. The kinetics of the whole-vacuole recordings: time-dependent activation and outward rectification point to SV channels (Fig. 6). In spite of using a gradient favouring Ca^2+^ release from the vacuole, only outward currents were registered (Fig. 6a, c). This confirms the recently formulated doubts concerning the role of SV channels in calcium signalling (Ranf et al. 2008). SV currents were also recorded when Mg^2+^ was the only cation in the medium and in the pipette (Fig. 6d, f). Again, the currents exhibited strong rectification and in the gradient promoting Mg^2+^ efflux to the cytosol no negative currents were registered. Note that in this case Ca^2+^ was not added to the bath and pipette solutions. Given that the residual Ca^2+^ content in the MgCl_2_ solution (in the absence of EGTA) is less than 1 μM, it was approx. at the threshold of SV activation (Fig. 4e). In addition, Mg^2+^ on the cytoplasmic side lowers the threshold of SV activation by Ca^2+^ and shifts the activation potential to more physiological (negative) values (Pei et al. 1999; Carpaneto et al. 2001).

Knowledge about the SV structure and structure–function relations greatly increased after identification of the SV channel protein (TPC1) (Peiter et al. 2005). TPC1 is a two-pore channel possessing two Ca^2+^ binding EF-hand motifs (Isayenkov et al. 2010). It was recently demonstrated that in *A. thaliana* the two EF-hands contribute largely to Ca^2+^-dependent SV channel activation. A crucial role in Ca^2+^ regulation was attributed to the EF-hand located near the 7th transmembrane loop (Schulze et al. 2011). The recognised genome of *Physcomitrella* provides an opportunity to find genes encoding TPC1. Thanks to blast research, we could determine the degree of similarity between TPC1 in *Physcomitrella* and another model organism—*A. thaliana*.

We checked the similarity of the TPC1 channel from *Physcomitrella* and from *Arabidopsis* in the public databases of genes and proteins (http://www.ncbi.nlm.nih.gov/). Encoded by the *PpTPC1* gene (GenBank accession AB211222), the putative TPC1 channel in *Physcomitrella* (BAF34917) is built of 752 amino acids and possesses two pore forming domains (amino acids 138–321; 484–684). A Blast (NCBI Blastp) search shows two putative TPC1 channels in *Arabidopsis* (NP_567258, BAB55460) with an almost identical protein length (733 amino acids) and 52 % sequence identity. Similar protein length (680–749 amino acids) and sequence identity (51–54 %) were exhibited by two-pore channels found in other plant species such as *Zea mays* (ACG26617), *Nicotiana tabacum* (Q75VR0), *Vitis vinifera* (XP_002273215, ACH53197, CBI21853), *Brachypodium distachyon* (XP_003569558), *Triticum aestivum* (Q6YLX9), *Arabidopsis lyrata* (XP_002874860), and *Populus trichocarpa* (XP_002330633).

Existence of calcium-binding domains in TPC1 from *Physcomitrella* was checked in the Prosite database of protein domains (http://prosite.expasy.org/). Calcium-binding domains were not found in the putative TPC1 channel (BAF34917) encoded by the *PpTPC1* gene, but was found in two other proteins from *Physcomitrella* (BAF34918, XP_001779439) similar to BAF34917 (64–65 % sequence identity).

In *Arabidopsis*, the VK channel is coded by the *TPK1* gene (Gobert et al. 2007). We have found in the NCBI database that *Physcomitrella* possesses proteins homologous to the product of *TPK1*, although these proteins are built of fewer amino acids than in *Arabidopsis* (363 amino acids in *Arabidopsis* and 291–317 in *Physcomitrella*). The sequence identity of the putative TPK1 proteins in *Physcomitrella* and *Arabidopsis* is also low and does not exceed 53 %. Again, like in the case of SV/TPC1, TPK1 in *Arabidopsis* possesses two EF-hand domains. This explains the requirement of submicromolar [Ca^2+^]_cyt_ for VK activation (Pottosin et al. 2003). In *Physcomitrella,* according to the Prosite database, one of the three putative TPK1 proteins possesses two EF-hand domains and the other two proteins—only one. Differences in the number of the EF-hand domains occur also in *Arabidopsis* TPK isoforms. For example, no EF-hands are present in AtTPK4 located in the plasma membrane (Becker et al. 2004). It is possible that in *Physcomitrella* different isoforms of the putative TPK1 are present and these isoforms exhibit different calcium dependence. It is also known that TPK1 in different species differ in respect to calcium dependence. In *Arabidopsis*, the minimum Ca^2+^ concentration which activates TPK1 is ≈0.5 μM (Gobert et al. 2007). In *Vicia faba*, activation of the VK channel probably coded by *TPK1* gene requires ≈1 μM cytoplasmic Ca^2+^ (Ward and Schroeder 1994). The effect of cytoplasmic Ca^2+^ was also examined in TPK1 from *Nicotiana tabacum*, which possesses only one EF-hand domain and is active in the absence of cytoplasmic Ca^2+^ (Hamamoto et al. 2008). Similar properties were observed in the case of the putative TPK1 channels from *Physcomitrella*, which were active at nearly 0 [Ca^2+^]_cyt_ (Fig. 5).

Our paper presents for the first time a survey of cation-permeable channels in the tonoplast of *Physcomitrella patens*. Future investigations with application of mutants will provide more detailed characteristics of the channels and help to disclose their physiological role.

## Abbreviations

SV: Slowly activating vacuolar channel
VK: Vacuolar K^+^ channel
FV: Fast activating vacuolar channel
E_K/Na/Ca/Mg_: Reversal potential for potassium/sodium/calcium/magnesium
[Ca^2+^]_cyt_: Cytoplasmic Ca^2+^ concentration
[Ca^2+^]_vac_: Vacuolar Ca^2+^ concentration
EGTA: Ethylene glycol tetraacetic acid
TPC: Two-pore channel
TPK: Two-pore K^+^ channel
